# Supplementary material for: Long Intergenic Noncoding RNA OIN1 Promotes Ovarian Cancer Growth by Modulating Apoptosis-Related Gene Expression
Source: Int J Mol Sci. 2021 Oct 18;22(20):11242. doi: 10.3390/ijms222011242 (PMC8541687; doi:10.3390/ijms222011242)
Supplement: Supplementary file 1 [file ijms-22-11242-s001.zip › ijms-1409600-supplementary.pdf]

Supplementary Materials

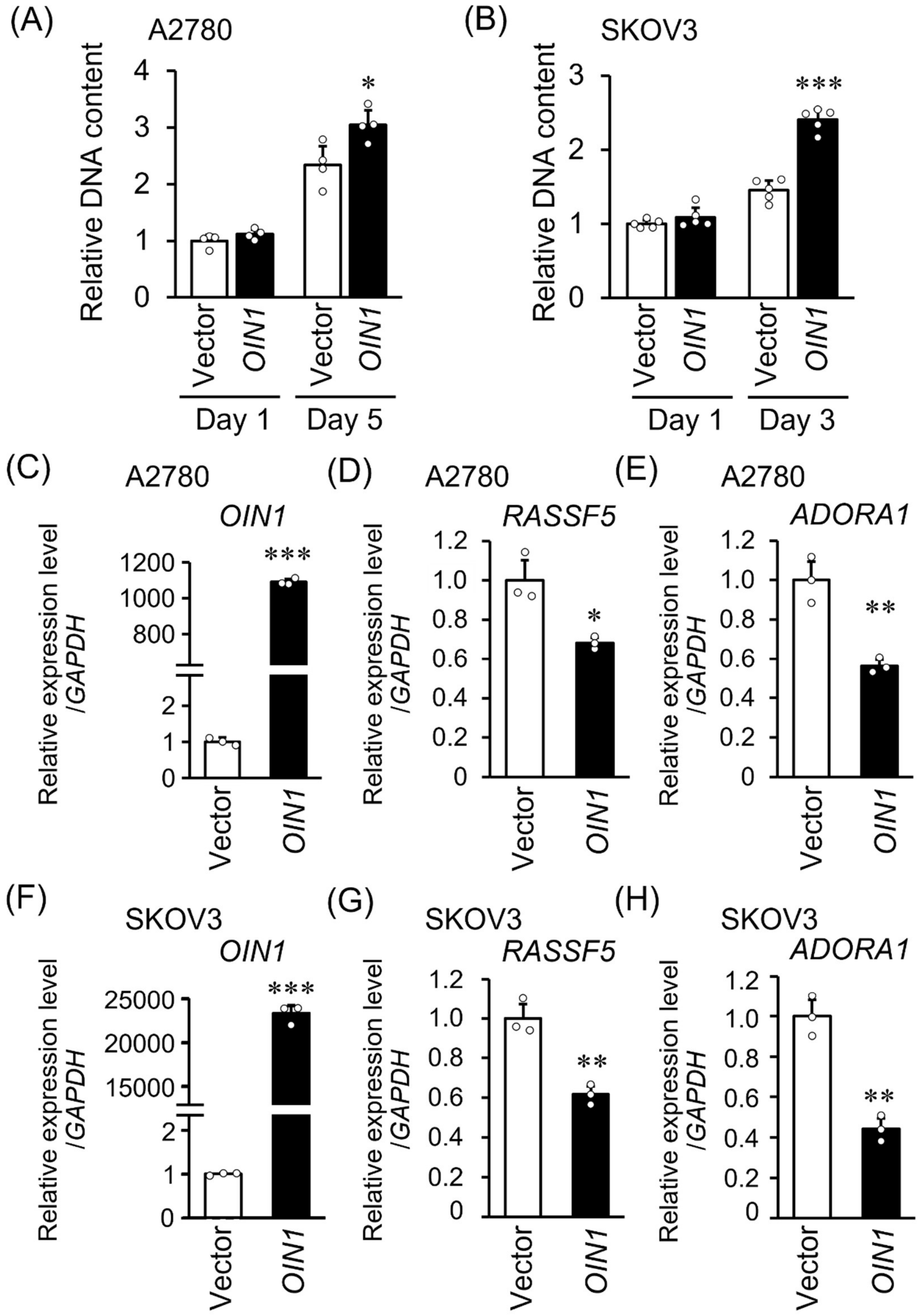

Figure S1. Exogenous *OIN1* expression induces cell proliferation and suppresses the expression of *OIN1* target genes in A2780 and SKOV3 cells. **A**,**B**, Proliferation of A2780 (**A**) and SKOV3 (**B**) cells transfected with *OIN1* expression or empty vector was analyzed using DNA assay. Data are presented as mean ± SD (A2780, $n = 4$; SKOV3, $n = 5$). **C**–**H**, Alteration of *OIN1* RNA (**C**,**F**) and *RASSF5* (**D**,**G**) and *ADORA1* (**E**,**H**) mRNA expression in A2780 and SKOV3 cells transfected with *OIN1* expression or empty vector. A2780 and SKOV3 cells were harvested 72 h and 24 h after plasmid transfection, respectively. Data are presented as mean ± SD ($n = 3$). *, $p < 0.05$; **, $p < 0.01$; ***, $p < 0.001$, Student's *t*-test.

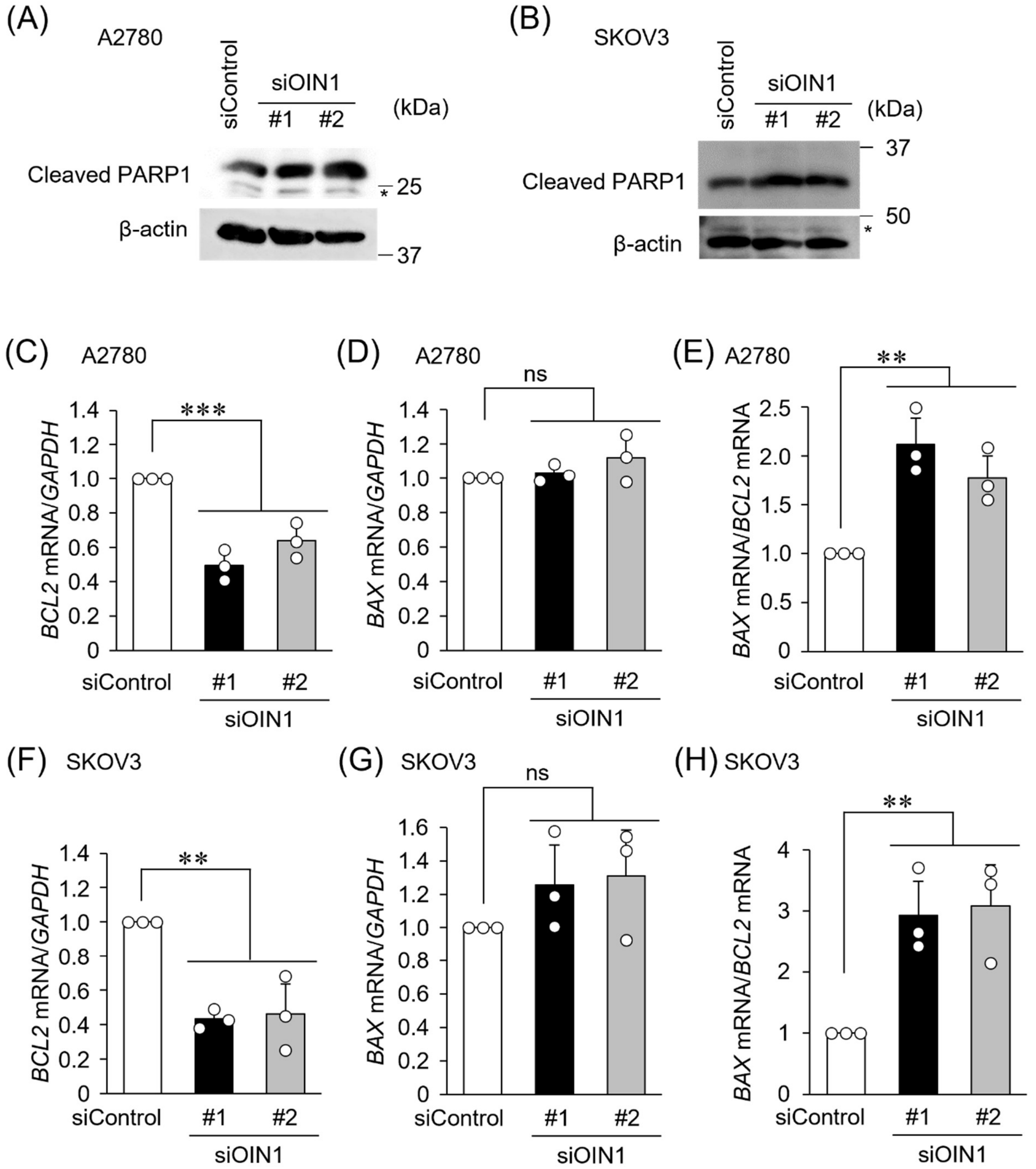

Figure S2. The expression levels of apoptosis-related factors in ovarian cancer cells in response to *OIN1* knockdown. **A**,**B**, cleaved PARP1 in A2780 (**A**) and SKOV3 (**B**) cells transfected with indicated siRNAs were analyzed by Western blotting. β-actin was used as a loading control. A2780 and SKOV3 cells were harvested 48 h and 72 h after siRNA transfection, respectively. *, non-specific band. **C–H**, Alteration of *BCL2* (**C**,**F**) and *BAX* (D,G) mRNA expression and the ratio of *BAX* mRNA to *BCL*2 mRNA in A2780 and SKOV3 cells transfected with indicated siRNAs. A2780 and SKOV3 cells were harvested 48 h after siRNA transfection. Data are presented as mean ± SD ($n = 3$). **, $p < 0.01$; ***, $p < 0.001$; ns, not significant, two-way ANOVA.

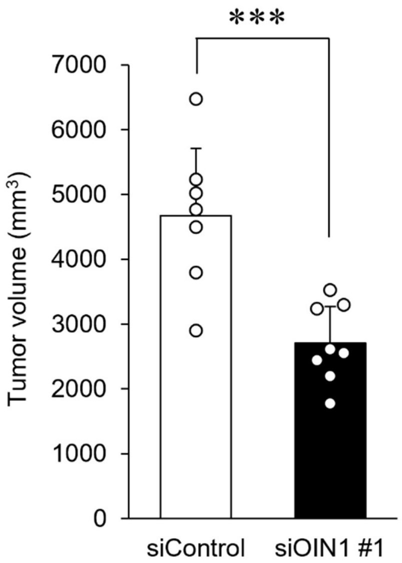

Figure S3. Tumor volume of female athymic mice xenografted with A2780 cells measured 18 days after the start of siControl or siOIN1 #1 injection, related with Figure 4B. Data are presented as mean ± SD (siControl, $n = 7$; siOIN1 #1, $n = 8$). ***, $p < 0.001$, Student's *t*-test.

Table S1. Positively correlated pathway genes with *OIN1* expression in ovarian cancer.

Term	Count	*p*-Value	Genes
Inner cell mass cell proliferation	4	3.90×10^{-4}	*BRCA2*, *GINS4*, *NCAPG2*, *TAF8*
DNA replication initiation	5	6.50×10^{-4}	*MCM2*, *MCM3*, *MCM5*, *PRIM2*, *GINS4*
Response to UV	5	1.80×10^{-3}	*ERCC6*, *MAPK8*, *MSH6*, *PMAIP1*, *USP1*
Response to X-ray	4	2.50×10^{-3}	*ERCC6*, *PMAIP1*, *BRCA2*, *XRCC2*
Positive regulation of viral genome replication	4	4.10×10^{-3}	*NOTCH1*, *PKN2*, *SRPK2*, *VAPB*
Amine metabolic process	3	4.10×10^{-3}	*AOC1*, *SULT1A1*, *SULT1C2*
Embryonic hindlimb morphogenesis	4	5.00×10^{-3}	*RPGRIP1L*, *NOTCH1*, *CHD7*, *WNT7A*
Cell cycle	9	6.10×10^{-3}	*RNF103-CHMP3*, *DAB2IP*, *E2F3*, *RABGAP1*, *JAG2*, *MCM2*, *CHMP3*, *PKN2*, *SUV39H1*
Sulfation	3	7.90×10^{-3}	*SULT1A1*, *SULT1A2*, *SULT1C2*
3'-phosphoadenosine 5'-phosphosulfate metabolic process	3	1.50×10^{-2}	*SULT1A1*, *SULT1A2*, *SULT1C2*

Table S2. Negatively correlated pathway genes with *OIN1* expression in ovarian cancer.

Term	Count	*p*-Value	Genes
Intracellular signal transduction	14	1.00×10^{-3}	*AKAP7*, *ARHGEF4*, *BLK*, *CISH*, *DGKA*, *GUCY2F*, *MAP3K12*, *MAST3*, *NOD1*, *NUAK2*, *PLCD1*, *PSEN2*, *RASSF5*, *STK32B*
Regulation of apoptotic process	7	4.10×10^{-2}	*RBM5*, *SARM1*, *NME3*, *QRICH1*, *RBM10*, *NOD1*, *RASSF5*
Cytoskeleton organization	6	4.20×10^{-2}	*WTIP*, *MAST3*, *NCKIPSD*, *PLD2*, *QRICH1*, *BLK*
Negative regulation of cell proliferation	10	4.50×10^{-2}	*RBM5*, *CDKN1B*, *WDR6*, *ADORA1*, *KIFAP3*, *DNAJB2*, *RARA*, *RBM10*, *BAP1*, *RASSF5*
Positive regulation of ligase activity	2	4.60×10^{-2}	*RIPK3*, *NHEJ1*
Regulation of protein stability	4	5.00×10^{-2}	*USP19*, *KAT2A*, *SIAH3*, *GNL3L*

Table S3. Primers used in qRT-PCR experiments.

Gene Name	Forward (5' to 3')	Reverse (5' to 3')
GAPDH	GGTGGTCTCCTCTGACTTCAACA	GTGGTCGTTGAGGGCAATG
OIN1	TCTTCACCCCTAACCAGCAGGAA	AGGACTGAAGTAAGTCCTGATGC
CDKN1B	TAATTGGGGCTCCGGCTAACT	TGCAGGTCGCTTCCTTATTCC
RASSF5	GGGCATGAAACTGAGTGAAGA	TGGCATCATAGATGGACTGGG
ADORA1	CCACAGACCTACTTCCACACC	TACCGGAGAGGGATCTTGACC
RBM5	ATGGGTTCAGACAAAAGAGTGAG	CTGCTTCGGGATTCACGCT
BAX	GTCGCCCTTTTCTACTTTGC	CTCAGCCCATCTTCTTCCAG
BCL2	AGTTCGGTGGGGTCATGTGTG	CTTCAGAGACAGCCAGGAGAAATC
